# Integrating Genomic Data with the Development of CRISPR-Based Point-of-Care-Testing for Bacterial Infections

**DOI:** 10.1007/s40588-024-00236-7

**Published:** 2024-10-15

**Authors:** Thanyapat Wanitchanon, Claire Chewapreecha, Chayasith Uttamapinant

**Affiliations:** 1 School of Biomolecular Science and Engineering, Vidyasirimedhi Institute of Science and Technology (VISTEC), Rayong, Thailand; 2 Mahidol Oxford Tropical Medicine Research Unit, Faculty of Tropical Medicine, Mahidol University, Bangkok, Thailand; 3 Department of Clinical Tropical Medicine, Faculty of Tropical Medicine, Mahidol University, Bangkok, Thailand; 4 Parasites and Microbe, Wellcome Sanger Institute, Hinxton, UK; 5 Centre for Tropical Medicine and Global Health, Nuffield Department of Medicine, University of Oxford, Oxford, UK

**Keywords:** Bacterial genomic, Diagnostic, CRISPR, Isothermal amplification, POCT

## Abstract

**Purpose of Review:**

Bacterial infections and antibiotic resistance contribute to global mortality. Despite many infections being preventable and treatable, the lack of reliable and accessible diagnostic tools exacerbates these issues. CRISPR (Clustered Regularly Interspaced Short Palindromic Repeats)-based diagnostics has emerged as a promising solution. However, the development of CRISPR diagnostics has often occurred in isolation, with limited integration of genomic data to guide target selection. In this review, we explore the synergy between bacterial genomics and CRISPR-based point-of-care tests (POCT), highlighting how genomic insights can inform target selection and enhance diagnostic accuracy.

**Recent Findings:**

We review recent advances in CRISPR-based technologies, focusing on the critical role of target sequence selection in improving the sensitivity of CRISPR-based diagnostics. Additionally, we examine the implementation of these technologies in resource-limited settings across Asia and Africa, presenting successful case studies that demonstrate their potential.

**Summary:**

The integration of bacterial genomics with CRISPR technology offers significant promise for the development of effective point-of-care diagnostics.

## Introduction

Bacterial infections remain a global public health burden. In 2019, out of 13.7 million infection-related deaths, 7.7 million were associated with 33 known bacterial pathogens, accounting for 13.6% of all global deaths [1]. Notably, 4.95 million of these deaths were linked to bacterial antimicrobial resistance [2]. The leading pathogens causing deaths were *Staphylococcus aureus*, *Escherichia coli*, *Streptococcus pneumoniae*, *Klebsiella pneumoniae*, and *Pseudomonas aeruginosa* [1]. For deaths associated with antimicrobial resistance, the bacterial list remains the same, with the addition of *Acinetobacter baumannii* [2]. Geographical and demographic variations significantly impact the outcomes of bacterial infections, with poorer results observed in lower- and middle-income countries (LMICs) compared to high-income countries (HICs). In Southeast Asia where most countries are classified as LMICs, neglected bacterial infections such as melioidosis, which causes an estimated 165,000 cases and 89,000 deaths annually, remain a significant health challenge [3]. Additionally, an increasing prevalence of third-generation cephalosporin-resistant *E. coli*, methicillin-resistant *S. aureus*, carbapenem-resistant *A. baumannii* and *K. pneumoniae* represent troubling trends in the region [4].

Several studies [5–7] have demonstrated that early detection of pathogens and their antibiotic resistance profiles, followed by effective treatment, can reduce treatment failures, improve clinical outcomes, and decrease mortality from bacterial infections. However, the limited capacity for microbiological diagnosis and analysis hinders early detection and appropriate treatment. LMICs often lack facilities or solely rely on traditional methods for culturing bacteria and performing conventional antimicrobial susceptibility testing (AST). While these methods are considered the gold standard for accurate disease and antibiotic resistance analysis [8, 9], they involve a lengthy process—typically requiring 3–14 days for bacterial growth in culture [10, 11], with additional time needed for AST [12] to determine effective antibiotics and appropriate treatment dosages.

Recent advancements in genomic data of bacterial pathogens and their genotype–phenotype profiles derived from genome-wide association studies [13] now allow for the identification of the causative pathogen and prediction of antibiotic resistance directly from genomic data. This precision biology holds promise in guiding treatment decisions, potentially reducing treatment costs linked with ineffective antibiotics. This approach may be particularly valuable in LMICs where resources are more limited. In parallel, recent advancements in molecular techniques [14] have increasingly focused on detecting pathogen nucleic acids to identify species and predict antibiotic susceptibility profiles. Nucleic acid amplification techniques (NAAT) such as polymerase chain reaction (PCR) [15], multiplex PCR [16, 17], reverse transcriptase PCR (RT-PCR) [18], PCR combined with restriction fragment length polymorphism (PCR–RFLP) [19], and real-time PCR (qPCR) [20–22], have been developed for pathogens with or without antibiotic resistance identification [23, 24]. However, these techniques still require thermal cyclers, which may not be readily available. Recent studies have pioneered the use of isothermal amplification methods like loop-mediated isothermal amplification (LAMP) and recombinase polymerase amplification coupled with CRISPR (RPA-CRISPR) for point-of-care diagnostics in LMICs [25–28]. These methods have demonstrated early success in the field, offering rapid, sensitive, and accurate results.

**Fig. 1:**
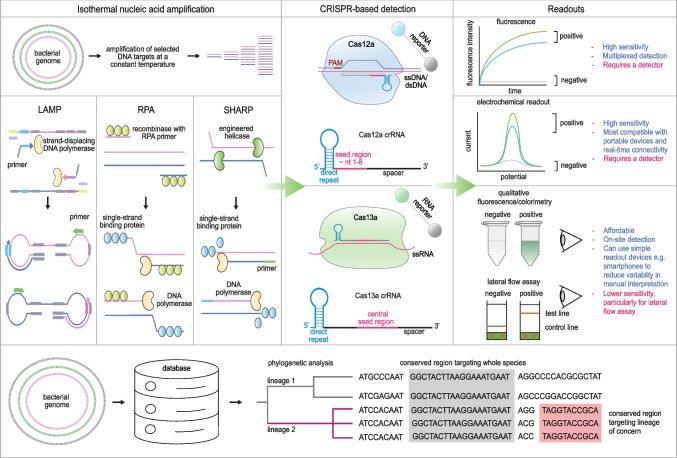
CRISPR-based diagnostics for point-of-care testing of bacterial infectious diseases. Top panel (left to right): Regions of interest within the bacterial genome can be isothermally amplified using techniques such as loop-mediated isothermal amplification (LAMP), recombinase polymerase amplification (RPA), and SSB helicase-assisted rapid PCR (SHARP). Amplicons can then be detected in a sequence-specific manner using crRNA-programmed DNA-targeted Cas12 or RNA-targeted Cas13 variants. For Cas13, in vitro transcription components are needed in the reaction, to convert DNA amplicons to RNA prior to detection. Diverse readouts of Cas-mediated detection are available, including quantitative fluorescence and electrochemical readouts and easily visualizable colorimetry and integration into lateral flow strips. Advantages and disadvantages of each readout are highlighted in blue and red, respectively. **Bottom panel**: A bacterial genomic database is used to identify conserved regions present in all strains of a species (highlighted in grey) and regions unique to specific lineages of concern (marked in pink)

This review summarizes common techniques and examples of CRISPR-based diagnostics for pathogenic bacterial detection, while emphasizing the potential benefits of applying genomic data for selection and design criteria for development of point-of-care testing by CRISPR-based diagnostics (Fig. 1).

## Nucleic Acid Amplification Without Thermal Cycling: Isothermal Amplification

PCR has been the gold-standard technology for nucleic acid amplification for genetic analyses and diagnostics including for the detection of bacteria. However, the requirement of thermal cycling of PCR can limit its application for resource-limited settings, point-of-care, or home uses [14]. To bypass the need of thermal cycling instruments, isothermal nucleic acid amplification approaches can be used. These techniques use enzymes—including strand-displacing polymerases, recombinases, and helicases—to facilitate nucleic acid strand separation instead of high temperatures needed in PCR, and can operate at single lower temperatures of 30–65 °C (Table 1). Such mesophilic incubation temperatures can be achieved by simple water baths, heat blocks, or ambient incubation in environmental and body temperatures.

**Table 1:** Characteristics of isothermal nucleic acid amplification reactions

Techniques	Temperature (°C)	Time (min)	Required proteins	Number of primers/target	Primer length (nt)	Amplicon size	Ref
Loop-mediated isothermal amplification (LAMP)	60–65	20–60	*Bst* DNA Polymerase	4–6	18–25	< 250	[29]
Recombinase polymerase amplification (RPA)	37–42	20–60	UvsX, UvsY, SSBP, Bsu DNA Polymerase	2	30–38	100–1,500	[30]
SSB-Helicase Assisted Rapid PCR (SHARP)	37–65	5–30	PcrA M6 Helicase, SSB, Bst-LF DNA polymerase, thermostable inorganic pyrophosphatase	2	18–23	155–6,000	[40]
Rolling circle amplification (RCA)	36	90–180	Ligase, strand-displacing polymerase	1	22 to 45	1,000–48,500	[41]
Exponential amplification reaction (EXPAR)	55–60	30	DNA polymerase, Nease	1 DNA trigger template	30	120	[42]
Strand displacement amplification (SDA)	30- 65	30–60	DNA polymerase, Nease	4	38–40	< 100	[43]
Strand-invasion based amplification (SIBA)	37–40	60	Recombinase, DNA polymerase	2 primers + 1 invasion oligo	16–23	< 1,000	[44]
Nucleic Acid Sequence Based Amplification (NASBA)	40–55	60–120	Avian myeloblastosis virus-reverse transcriptase (AMV-RT), RNase H, and T7 RNA polymerase	2	17–29	100–250	[45]
Cross-priming amplification (CPA)	59–65	60–90	*Bst* DNA polymerase	5	18–53	150–200	[46]

The most widely used techniques for isothermal amplification are loop-mediated isothermal amplification (LAMP) [29] and recombinase polymerase amplification (RPA) [30] (Fig. 1). Both techniques amplify nucleic acids exponentially and can achieve sensitivity approaching PCR in 20–60 min [31–33]. LAMP uses 4–6 primers capable of forming loops, which can be extended by strand-displacing DNA polymerases such as a thermostable *Bacillus stearothermophilus* (Bst) DNA polymerase I [32]. LAMP works well at 60–65 °C, is highly sensitive and has commercially available reagents, but the amplification can be non-specific unless coupled with sequence-specific detection methods such as CRISPR. LAMP also generates large, concatemeric amplicons that are well-known to resist degradation and carry over in testing environments, creating false positive results. The contamination problem can be mitigated by coupling LAMP with uracil DNA glycosylase (UDG) [34], which creates non-amplifiable abasic sites on DNA from previous amplification events.

RPA uses DNA recombinases such as *T4* or *T6* UvsX recombinase capable of forming sequence-specific strand-displacing complexes with primers [30]. The resulting strand exchange complex is stabilized by single-stranded binding proteins such as *T4* Gp32, allowing DNA polymerization by strand-displacing polymerases to occur. Due to assistance from recombinase-mediated strand exchange, RPA can operate at lower temperatures (37–42 °C) than LAMP. RPA primers are also much less complex than LAMP primers and its amplicons much smaller, making multiplexed amplification by RPA more straightforward and the technique less prone to carryover contamination compared to LAMP. However, RPA has complex formulations, with only two companies managing its global supply, making its prospect for use in routine diagnostics more limited. Similar to LAMP, RPA can be prone to non-specific amplification, necessitating coupling to sequence-specific detection modalities such as hybridization-based probes or CRISPR to boost up its specificity.

Molecular components of LAMP and RPA techniques have continually been improved to achieve the diagnostic accuracy level of PCR while preserving their point-of-care attributes. Engineered forms of Bst polymerase [35] are considered state-of-the-art for LAMP, and deeper insight into kinetics of steps during the LAMP reaction has identified strand invasion during initiation as rate-limiting [36], paving way for further engineering of the reaction. Similarly, the strand invasion by recombinases has been identified as the rate-limiting step for RPA [37], opening opportunities for discovery and engineering of recombinases to improve the reaction. To facilitate adoption of the technologies, automated primer design tools such as NEB LAMP [38] and PrimedRPA [39] are available.

Beyond LAMP and RPA, another fast and accurate isothermal amplification technology is SSB-Helicase Assisted Rapid PCR (SHARP) [40]. SHARP uses a thermophilic PcrA helicase engineered to increase unwinding activity, can generate linear amplicons of diverse sizes akin to PCR within 30 min, and operates at 37–65 °C. Characteristics of other common isothermal amplification techniques are shown in Table 1.

## CRISPR-Based Diagnostics for the Identification of Bacterial Pathogens

Clustered regularly interspaced short palindromic repeats (CRISPR) and their protein effector CRISPR-associated (Cas) enzymes are adaptive immune systems found in bacteria, archaea, and certain viruses, protecting the hosts against invading genetic elements such as bacteriophages and plasmids. The key programmable component, CRISPR RNA (crRNA), ensures specificity by recognizing targets based on the unique structure of crRNA, which includes a specific length and orientation of direct repeats (DR) and spacers for each Cas enzyme type. This recognition allows for precise cutting of the target (*cis* activity) and nearby nucleic acids (*trans* activity), enabling accurate sequence-specific editing and detection. Tools for accelerated design of crRNAs—such as PrimeSHERLOCK [47], CHOPCHOP [48], CRISPRdirect [49], CaSilico [50], DeepCRISPR [51], GuideMaker [52], CRISPRscan [53], CRISPR-DT [54], benchling [55] and pathoGD [56]—are now readily available. PathoGD, in particular, has begun incorporating genomic databases into its target design. However, this remains an evolving area as genomic databases for many species continue to expand.

Among CRISPR-Cas systems, class 2 systems—composed of a single CRISPR RNA (crRNA)-binding protein—are more widely used for both gene editing and detection applications, as they are simpler to produce and use. Within class 2 systems, type V (Cas12) and type VI (Cas13) are primarily used in diagnostic applications [57, 58], as they possess “collateral” non-specific cleavage activity which massively amplify detection signal (generally obtained through cleavage of bystander nucleic acid probes) upon target recognition. CRISPR-based diagnostics have nanomolar-picomolar sensitivity levels by themselves, but upon coupling to isothermal amplification techniques like LAMP and RPA, can reach attomolar sensitivity levels on par with PCR. Amplification-free Cas-based detection is not sensitive enough to detect low-concentration pathogens, but could be useful in cases where pathogen loads may not be an issue, such as in highly concentrated sputum samples. To enhance the limit of detection in amplification-free CRISPR diagnostics, selection of multiple nucleic acid targets or targets with multi-copy/repeating gene units such as IS6110 and IS1081 for detection of MTBC [59, 60] would help compensate for the signal loss from the lack of nucleic acid amplification.

An important consideration when using CRISPR-based detection is the choice of the Cas systems. In an isothermal amplification-coupled CRISPR diagnostic assays, both DNA-targeting Cas12 and RNA-targeting Cas13 systems can be used to detect generated amplicons. On one hand, Cas12 can detect DNA amplicons without reverse transcription requirement but requires the presence of a protospacer adjacent motif (PAM) next to the target DNA for maximal detection efficiency [61]. Recent findings show PAM-less detection is nevertheless possible [62]. To detect single nucleotide polymorphisms (SNPs), Cas12f, which requires sequence complementarity in the seed region for target single-stranded DNA recognition, is an enzyme of choice for optimal specificity [63]. An engineered *Lachnospiraceae bacterium* Cas12a (LbCas12a) with enhanced sensitivity to distinguish SNPs has also been reported [64]. Target specificity can also be improved through the use of chimeric DNA-RNA guides [65]. Thermostable Cas12 variants enable one-pot formulations of CRISPR-based detection with LAMP amplification [66, 67].

On the other hand, RNA-targeting Cas13 can detect RNA targets without PAM requirements, permitting more facile selection of target sites for detection than Cas12. Among Cas enzyme variants typically used in genetic detection, *Leptotrichia wadei* Cas13a (LwaCas13a) has been shown to possess the highest collateral activity levels and generate detection signals with the fastest kinetics [68]. To detect DNA amplicons generated from isothermal amplification with Cas13, DNA amplicons are first transcribed into RNA via RNA polymerases such as T7 RNA polymerase (T7 RNAP). This added T7 RNAP step was shown to further enhance the overall sensitivity of the detection [69]. Further engineering efforts to increase general affinity for RNA substrates, such as the insertion an RNA-binding loop to the HEPN domain of LwaCas13a, can improve the sensitivity of Cas13-based detection [70]. Different Cas13 variants can have distinct dinucleotide cleavage preferences and can be programmed to detect different sequences in an orthogonal manner in a single reaction [71]. Such multiplexed Cas-based detection with orthogonal Cas13 enzymes can work in conjunction with Cas12 to generate up to four-channel, single-reaction detection [71]. Similar to Cas12, there are now thermostable Cas13 variants which enable genetic detection at higher temperatures, with higher specificity [72].

Examples of CRISPR-based technologies for the identification of globally relevant bacterial infection are shown in Table 2.

**Table 2:** CRISPR-based diagnostics for clinically important bacteria

Pathogen	Effector Cas	Target	Amplification method	Source of samples	Readout	LOD	Approximate time to perform the test, excluding sample collection and nucleic acid extraction steps). For examples marked with *, time given is from sample preparation to obtaining results	Sensitivity/specificity with number of clinical samples in parentheses	Ref
*B. pseudomallei*	Cas12a	Conserved region	RPA	Peripheral blood, respiratory secretion fluids, urine, pus or other body fluids	LFA	50 CFU/ml	2.3 h for urine*	93.0% sensitivity (103 of 114)	[25]
							3.3 h for respiratory secretion, pus and other body fluids*	96.8% specificity (209 of 216)	
							1.1 days for blood sample*		
*M. tuberculosis* complex (MTBC)	EnGen Lba Cas12a	IS6110	PCR / RPA	Serum	F (PCR- CRISPR-TB),	0.06 copy/µl (PCR CRISPR-TB)	1 h (PCR CRISPR-TB)	PCR-CRISPR-TB	[73]
					LFA (RPA-CRISPR-TB)		30 min (RPA-CRISPR-TB)	96% sensitivity(27 of 28)	
								94% specificity (16 of17) in adult cohort	
								83% sensitivity(5 of 6)	
								95% specificity (21 of 22) in children cohort	
*M. tuberculosis* complex (MTBC)	LwaCas13a	IS6110 and/or IS1081	Pooled PCR/ RPA	Plasma-derived cell-free DNA	F (WATSON)	0.01–0.1 GE/reaction (WATSON)	4 h (WATSON)	91% sensitivity (10 of 11 using WATSON) in samples from South Africa	[28]
					LFA (PCR/RPA-13a)	~ 0.05 GE/reaction (WATSON-IS1081)	30 min (RPA—13a-LFA)	89% sensitivity (8 of 9 using WATSON) in samples from Uganda	
						0.1 – 1 GE/ reaction (PCR/RPA-13a-LFA)		100% specificity (no cross reactivity in NTM and the other common bacterial pathogens)	
*M. tuberculosis* complex (MTBC)	Cas12a	IS6110	RPA	Bronchoalveolar lavage fluid, hydrothorax,homogenate of needle biopsy	F, LFA	1 copy/µl(F)	40 min (F)	80% sensitivity (F)	[74]
								100% specificity (F)	
								in 69 clinical samples	
Methicillin-resistant	Cas12a/	*mecA* and *clfA* genes	Duplexed RPA	Fester, sputum, urine	F	5 copies/µl	30 min	100% sensitivity (18 of 18 MRSA and 3 of 3 MSSA)	[75]
*S.aureus* (MRSA)	Cas13a							100% specificity (5 of 5)	
Carbapenem-resistant *K. pneumoniae* (CRKP)	Cas13a	*KP* and *bla*_*KPC*_ and *bla*_*NDM*_ gene	PCR/RAA	Cultured bacteria	F, LFA	1 copy/µl (PCR-CRISPR-F)	3 h	Sensitivity: N/A	[76]
						10 copies/µl (RAA-CRISPR-F/RAA-CRISPR-LFA)		Specificity: N/A	
Carbapenem-Resistant Enterobacterales	Cas13a	*bla*_*KPC*_	RPA	Sputum	F	2.5 copies/µl	1 h	97.14% sensitivity (102 of 105)	[77]
								100% specificity (206 of 206)	
Carbapenem-resistant *A. baumannii*	Cas13a	*OXA-51* and *OXA-23* genes	Duplexed RPA	Clinical strains	F	0.3 copy/µl (1.3 × 10^−6^ ng/μl)	1.5 h	100% sensitivity (22 of 22) for CRAB strain	[78]
								100% sensitivity (22 of 22) for AB strain	
								100% specificity (21 of 21)	
*N. gonorrhoeae, C. trachomatis*	Cas12a/Cas13a	*porA* and cryptic plasmid fragment	Duplexed RPA	Urine	F	1 copy/µl for both pathogens	75 min	*N. gonorrhoeae*	[79]
								100% sensitivity (33 of 33)	
								100% specificity (35 of 35)	
								*C. trachomatis*	
								84.85% sensitivity (28 of 33)	
								100% specificity (55 of 55)	
								Co-infection	
								84.62% sensitivity (11 of 13)	
								100% specificity (75 of 75)	
10 species for pneumonia	Cas12a	species-specific DNA tags	PCR	BALF from pneumonia patients	F	N/A	4 h	~ 100% Sensitivity	[80]
								~ 87% Specificity	
*P. aeruginosa*	EnGen Lba Cas12a	*oprL* gene	RPA	BALF	F	8 copies/reaction	30 min	100% sensitivity (19 of 19)	[81]
								100% specificity (77/77)	
*Salmonella enterica*	LbCas12a	*parC* gene with S80I mutation	PCR	Stool	F	1 copy/reaction	3 h	100% sensitivity (28 of 28)	[82]
								98.48% specificity (65/66)	
*Shigella flexneri*	Cas12a	Highly conserved hypothetical gene	LAMP	Clinical isolates	F	4 copies/μl	40 min	100% sensitivity (20 of 20)	[83]
								Clinical specificity not available but no cross reactivity in selected non-*S. flexneri*	

* LOD* Limit of Detection. *RPA* Recombinase Polymerase Amplification. *RAA* Recombinase-Aided Recombination. *F* Fluorescence. *LFA* Lateral Flow Assay. *WATSON* Whole-genome Assay using Tiled Surveillance Of Nucleic acids. *GE* Genome Equivalent. *MRSA* Methicillin-Resistant *Staphylococcus aureus. MSSA* Methicillin-Sensitive *Staphylococcus aureus. BALS* bronchoalveolar lavage fluid. *LAMP* Loop-mediated isothermal Amplification. *N/A* Not assessed. *CRAB* Carbapenem-resistant *Acinetobacter baumannii. AB Acinetobacter baumannii*

## Utilizing Genomic Data to Improve the Sensitivity and Specificity of CRISPR-Based Diagnostics

Developing a successful nucleic acid-based diagnostic tool relies on the careful selection of target sequences within bacterial genomes. These sequences must be complementary to both primers used for target amplification and the crRNA that guides a Cas enzyme to specific targets. While the design of primers for DNA amplification methods has been intensively reviewed [32, 33, 84, 85], the design of target sequences that complement crRNA has not been widely discussed and is a key focus here.

The crRNA consists of two main components: a direct repeat and a spacer sequence. The direct repeat is essential for maintaining the structural integrity of crRNA and facilitating its binding to the Cas enzyme, while the spacer sequence is crucial for recognizing and targeting specific nucleic acids that identify bacterial species, lineages, or genes of interest. The spacer sequence must be complementary to the specific target in the bacterial genomes. A typical spacer sequence ranges from 20 to 28 base pairs and includes a seed region critical for initiating base pairing with the target nucleotide sequence. The structure of the seed region varies depending on the chosen Cas enzyme. For LbCas12a, the seed region is located within the first eight nucleotides of the crRNA spacer (nucleotide 1—8) [86, 87] and requires an additional protospacer adjacent motif (PAM) sequence adjacent to the 5’end of the crRNA spacer [88]. In contrast, the seed region for LwaCas13a is found between the central region of crRNA spacer and does not require a PAM sequence, unlike Cas12 systems [89]. The remainder of the spacer should also maintain complementarity to the target bacterial sequence to ensure stable and effective binding.

To enhance sensitivity, the target sequence in the bacterial genomes should be present in the majority, if not all, strains within the lineage or species of interest. However, designing targets based solely on the presence of sequences in a few reference genomes often leads to suboptimal results, particularly for species with high genetic diversity. For instance, nucleic acid-based detection of *Mycobacterium tuberculosis*, a species with a highly clonal population, achieves relatively good sensitivity [73, 90]. In contrast, species with high recombination rates and frequent gene flow, such as the *S. pneumoniae* [91, 92], *B. pseudomallei* [93]*, N. gonorrhoeae* [94], *or Enterobacteriaceae* [95–97], present significant challenges. Targets located in recombination hotspots or hypermutating regions [98–100] such as genes interacting with the host, are likely to mutate to evade host immune detection, which can diminish the sensitivity of tests as these pathogens continue to evolve.

Population-level genomic data are crucial for identifying conserved non-hypervariable regions across diverse populations. Currently, public databases house over two million bacterial and 16 million viral genomic entries (data retrieved on 11 August 2024 from NCBI and GISAID). However, more than 90% of this data is concentrated on a select few bacterial and viral pathogens, representing less than 1% of the total species [101]. Notable examples include *S. enterica*, *E. coli*, and *S. pneumoniae* for bacteria, and SARS-CoV-2 for viruses. Despite discrepancies in species representation, genomic data can be leveraged to identify relevant targets for specific genes and lineages among well-studied species. Several tools have been developed to identify conserved regions by extracting “core” genes from the pan-genome [102] or K-mer-based analysis [103, 104], which is promising for identifying species-specific target sequences. For lineage-specific targets, a robust classification system is required. Phylogenetic trees are commonly used to determine lineage structure [105]. Genetic variations, such as single nucleotide polymorphisms (SNPs), insertions, or deletions, can be mapped onto the nodes of the phylogenetic tree. Genetic variations reconstructed to the ancestral node of the lineage of interest, and hence carried in all strains belonging to that lineage, can be selected as targets [106]. Tools for bacterial genome-wide association studies (GWAS) have been developed to identify genes or genetic variants associated with antibiotic susceptibility or virulent traits [107, 108] in organisms like *M. tuberculosis* [109, 110], *S. pneumoniae* [111], and *K. pneumoniae* [96, 112, 113], *N. gonorrhoeae* [114–116], and *B. pseudomallei* [117]. Together, these approaches facilitate the selection of clinically relevant targets with enhanced sensitivity.

To ensure specificity, the target sequences must not overlap with those of other species, including other pathogens or the human host. The target sequences identified in the first step can be filtered based on the number of mismatches with non-target sequences archived in public databases, which serves as an initial in silico screening. The tolerance of CRISPR-Cas systems for mismatches between the crRNA and target bacterial sequences varies depending on the specific Cas enzyme. Cas12 typically tolerates a few mismatches within the spacer region; however, mismatches in the seed region are more detrimental and can affect the binding efficiency [118]. In contrast, Cas13 generally exhibits a higher tolerance for mismatches [119], though the exact tolerance may vary based on the specific Cas13 variants and the target nucleotides. It is also important to consider the clinical context and the presence of other bacterial species or competing targets in the same setting to ensure the specificity of CRISPR-based diagnosis. Additionally, the assay production pipeline must be taken into account, particularly since *E. coli* is used to synthesize the enzymes required for CRISPR assays. Designing target sequences for pathogenic *E. coli* can be challenging because *E. coli* strains are used to manufacture the necessary protein components of the test. To minimize false positives caused by residual DNA bound to the enzyme, target sequences can be designed to cover regions absent in the manufacturing *E. coli* strain, such as the capsular region [120, 121]. These considerations can minimize off-target effects thereby improving the specificity of the assays.

## Real-World Applications

Over the past few years, we have seen significant progress in translating CRISPR-based diagnostics to clinical practice for a variety of bacterial pathogens (Table 2). These advancements have been particularly impactful in resource-limited settings across the global south with examples including the use of CRISPR-based diagnostics to address both endemic diseases such as melioidosis, and global challenges, such as tuberculosis.

Melioidosis, a severe bacterial infection caused by *B. pseudomallei* [122], is a public health concern in many tropical and sub-tropical countries [122–125]. Southeast Asia [126–129], particularly northeast Thailand [130], is considered a global hotspot for this disease. The bacterium is commonly found in contaminated soil and water in endemic areas. Serological studies [131] have shown that healthy individuals in these regions frequently encounter the bacterium, resulting in high antibody titers that limit the effectiveness of immunology-based diagnostic tests. Once infected, the mortality rate can be as high as 40% [132, 133]. The current gold standard for melioidosis is culture confirmation, which has a reported low sensitivity and requires 3–4 days for a confirmed diagnosis [134]. A study conducted in northeast Thailand found that 22% of melioidosis patients died before receiving a culture-confirmed diagnosis, and 26% of those who survived until the culture results were available died within 28 days of admission [25]. The survival rate was significantly higher when patients received the correct antibiotics on the first day of admission, highlighting the urgent need for rapid, point-of-care diagnostic tests.

To address this need, a CRISPR-Cas12a-based diagnostic test called CRISPR-BP34 has been developed and implemented in a hospital located in northeast Thailand [25]. This assay has been applied directly to various clinical sample types, including blood, urine, sputum, respiratory secretion, pus and other body fluids. CRISPR-BP34 has a sensitivity of 93.0% (106 of 114 samples), compared to 66.7% (76 of 114) for culture. Importantly, CRISPR-BP34 significantly reduced the time to diagnosis, with a sample-to-diagnosis time of 1.1 days for blood samples, 2.3 h for urine, and 3.3 h for respiratory secretion, pus and other body fluids. This rapid diagnostic capability allows for quicker administration of the correct treatment, potentially saving more lives from melioidosis.

Tuberculosis (TB) presents a significant global health challenge [135]. The disease displays diverse clinical manifestation ranging from latent TB, characterized by asymptomatic bacterial carriage; to active TB, where symptoms such as persistent cough, fever, and weight loss often overlap with other diseases [136–139]. The diagnostic gold standard, culturing *M. tuberculosis*, is hindered by the bacterium’s slow growth rate, resulting in prolonged time to diagnosis—often weeks [140]. Furthermore, this method requires sputum samples, which are difficult to obtain from patients with extrapulmonary TB or those who are severely ill, thereby limiting its diagnostic utility. Molecular diagnostics, such as the GeneXpert MTB/RIF assay, have been developed to detect *M. tuberculosis* DNA and rifampicin resistance-associated mutations in the *rpoB* gene [141–143]. Despite their utility, these assays still require samples with high bacterial loads, predominantly from sputum, and necessitate infrastructure like stable electricity and temperature control, making them less feasible in resource-limited settings.

Recent developments in CRISPR-based diagnostics, including the CRISPR-Cas12a system (CRISPR-TB) [73] and the multiplex CRISPR-Cas13 system (WATSON) [28], have demonstrated enhanced sensitivity by targeting repetitive insertion elements. CRISPR-TB targets IS6110 (present in 1–25 copies per genome) [144], while WATSON targets both IS6110 and IS1081 (present in 5–7 copies per genome) [59]. These systems enable the detection of *M. tuberculosis* DNA from non-sputum samples, such as cell-free DNA (cfDNA) in blood or serum, addressing the limitations of conventional methods. Empirical data from these studies underscore the efficacy of these CRISPR-based diagnostics. CRISPR-TB exhibited a sensitivity of 96% (27 of 28 samples) in HIV-positive adults in Eswatini—a risk group known for lower bacterial loads—and achieved 100% sensitivity (13 of 13) in Kenyan patients. Similarly, WATSON reported 91% sensitivity (10 of 11) in samples from South Africa and 89% sensitivity (8 of 9) in samples from Uganda.

The high sensitivity, rapid turnaround, and minimal equipment requirements of these CRISPR-based assays position them as strong candidates for point-of-care diagnostics, particularly in resource-limited settings where traditional diagnostic infrastructure may be lacking.

## Extending CRISPR-Based Technologies to Point-of-Care Testing

With sufficient input from bacterial genomic information aiding the selection of appropriate test targets, CRISPR-based technologies fundamentally possess very high diagnostic accuracy attributes to meet WHO’s REASSURED criteria [145]. Continual improvements to improve other aspects of REASSURED diagnostic tests—such as ease of specimen collection, rapidness and robustness of tests, simplified test procedure and equipment for testing, and real-time connectivity—would make CRISPR-based tests truly useful for pathogenic bacterial detection in resource-limited settings of LMICs.

To reduce the time from sample collection to obtaining results and to make testing more appropriate for point-of-care use, strategies to bypass machine-aided nucleic acid extraction are continually developed. Simple lysis buffer recipes compatible with downstream amplification and CRISPR-based detection along with heat inactivation are shown to work well with viruses [146], bacteria and even parasites [147]. Since CRISPR-based detection relies on Cas-mediated cleavage of nucleic acid reporters, care must be taken to ensure buffer recipes and lysis procedure completely inactivate nucleases which may otherwise cause both false positives (from non-specific cleavage of reporters) and false negatives (from degradation of nucleic acid input). A built-in endonuclease reporter capable of distinguishing non-specific RNase-mediated cleavage from specific Cas-mediated cleavage can be used to inform on potential endonuclease contamination in samples or the testing environment [148]. Beyond buffer recipes, nanoscale magnetic beads [149], membranes [150, 151], and microfluidic platforms [152, 153] can be used for quick nucleic acid extraction prior to amplification and detection.

To simplify test procedure and reduce the risk of carryover contamination, one-pot formulations where components for nucleic acid amplification and CRISPR-based detection can be pre-mixed are preferred over sequential amplification then CRISPR-based detection. Configuring one-pot formulations of nucleic acid amplification and CRISPR-based detection while maintaining similar diagnostic accuracy as sequential reactions have been successful for LAMP-based amplification [154–157] but have proven trickier for other isothermal amplification reactions including RPA, likely due to complexity and specific conditions needed for the amplification reaction. Having active Cas-mediated *tran*s-cleavage in the same tube as an isothermal amplification can also exert a negative effect, due to undesired cleavage of nucleic acid templates or primers needed for the amplification by activated Cas enzymes. To this end, innovative probe designs such as photoactivatable crRNAs can be used to turn on Cas activity after nucleic acid amplification is already completed, while still allowing all reaction components to be in the same tube for easy handling [158, 159].

Amplification-free CRISPR-based detection is intrinsically a simpler procedure and has lower contamination risks upon use, but possesses lower diagnostic accuracy than amplification-coupled variants. Its accuracy can be improved through coupling with ultra-sensitive readout systems such as electrochemical readouts [160]. Feedback signal amplification can also be exploited through the use of crRNAs that once cleaved, can feedback-activate Cas-based detection for signal amplification [161]. The ability of amplification-free Cas13-based detection in quantifying RNA levels may be useful in differentiating basal vs overexpression levels of inducible antibiotic resistance determinants.

For multiplexed detection, up to four targets of nucleic acid can be simultaneously detected in the same tube via orthogonal Cas enzymes [162]. This strategy provides valuable information for diagnosis and timely treatment for diseases which present similar symptoms and reduces the cost of detection per sample. However, optimizations for simultaneous isothermal amplification and CRISPR-based detection of multiple targets are required to boost the limit of detection while maintaining specificity [163]. Further increase of detection targets to more than 4,500 regions is possible using single-chip multiplex digital droplet assay platforms such as CARMEN [164, 165], which can massively reduce reagent volume used down to nanoliters. However, such platforms come at a cost of expensive and complicated instrumentation.

To reduce ambiguity in manual interpretation and enhance usability and real-time connectivity, several studies have pioneered the design of the readout system that can be automatically detected and transfer data through portable devices, such as mobile phones [166–168] and personal glucose meters [169, 170]. This approach offers promising options for enabling point-of-care detection of pathogenic bacteria.

## Conclusions

As genomic databases of bacterial pathogens continue to expand, test developers can leverage such wealth of information to precisely design point-of-care nucleic acid tests for bacterial species, lineages, or antibiotic-resistant genes of interest. CRISPR-based diagnostics combine the high sensitivity of nucleic acid amplification techniques with sequence-specific Cas-based detection, can detect single-nucleotide differences in sequences as well as large deletions or insertions, and can be multiplexed—thus possessing many attributes which could transform point-of-care testing of bacterial infectious diseases. In addition to developments to testing platforms suitable for point-of-care use, concurrent developments of high-throughput approaches to test primers and crRNAs and machine-learning algorithms to process these large datasets will ultimately yield robust design tools for CRISPR diagnostics which can be adapted for any pathogen target. Beyond use in rapid diagnosis to inform patient treatment options, field-deployable CRISPR diagnostics could facilitate continuous monitoring of infectious agents in the environment, which could lead to improved sanitation practices or timely management of (re)emerging infectious diseases.

## Key References

- Chen JS, Ma E, Harrington LB, Da Costa M, Tian X, Palefsky JM, et al. CRISPR-Cas12a target binding unleashes indiscriminate single-stranded DNase activity. *Science*. 2018;360(6387):436–9.
  - ∘ A seminal study describing CRISPR-Cas12-based detection of nucleic acids.
- Gootenberg JS, Abudayyeh OO, Lee JW, Essletzbichler P, Dy AJ, Joung J, et al. Nucleic acid detection with CRISPR-Cas13a/C2c2. *Science*. 2017;356(6336):438–42.
  - ∘ A seminal study describing CRISPR-Cas13-based detection of nucleic acids.
- Gootenberg JS, Abudayyeh OO, Kellner MJ, Joung J, Collins JJ, Zhang F. Multiplexed and portable nucleic acid detection platform with Cas13, Cas12a, and Csm6. *Science*. 2018;360(6387):439–44.
  - ∘ A seminal development of multiplexed detection based on orthogonal Cas enzymes and signal amplification strategies.
- Blackwell GA, Hunt M, Malone KM, Lima L, Horesh G, Alako BTF, et al. Exploring bacterial diversity via a curated and searchable snapshot of archived DNA sequences. *PLOS Biology*. 2021;19(11):e3001421.
  - ∘ A paper providing a comprehensive summary of the resources available for population-scale bacterial genomics, offering a searchable database that enables the investigation of specific genes, mutations, and plasmid
- Myhrvold C, Freije CA, Gootenberg JS, Abudayyeh OO, Metsky HC, Durbin AF, et al. Field-deployable viral diagnostics using CRISPR-Cas13. *Science*. 2018;360(6387):444–8.
  - ∘ The development of a field-deployable platform for CRISPR-based diagnostics.
- Ackerman CM, Myhrvold C, Thakku SG, Freije CA, Metsky HC, Yang DK, et al. Massively multiplexed nucleic acid detection with Cas13. *Nature*. 2020;582(7811):277–82.
  - ∘ The development combining CRISPR-based detection with microfluidic-based reaction miniaturization, enabling massively multiplexed detection of pathogens.
- Patchsung M, Jantarug K, Pattama A, Aphicho K, Suraritdechachai S, Meesawat P, et al. Clinical validation of a Cas13-based assay for the detection of SARS-CoV-2 RNA. *Nat Biomed Eng.* 2020;4(12):1140–9.
  - ∘ Large-scale validation and demonstration of utility of CRISPR-based diagnostics for SARS-CoV-2 in a LMIC.
- Pakdeerat S, Boonklang P, Angchagun K, Chomkatekaew C, Apichaidejudom N, Dokket Y, et al. Benchmarking CRISPR-BP34 for point-of-care melioidosis detection in low-income and middle-income countries: a molecular diagnostics study. *The Lancet Microbe*. 2024;5(4):e379-e89.
  - ∘ Demonstration of utility of CRISPR-based diagnostics for faster diagnosis of melioidosis in a LMIC, which could lead to faster initiation of life-saving treatment.
- Thakku SG, Lirette J, Murugesan K, Chen J, Theron G, Banaei N, et al. Genome-wide tiled detection of circulating *Mycobacterium tuberculosis* cell-free DNA using Cas13. *Nature Communications*. 2023;14(1):1803.
  - ∘ Development of CRISPR-based WATSON to detect low amount of cell-free DNA, enabling detection of M. tuberculosis in plasma samples.
- Huang Z, LaCourse SM, Kay AW, Stern J, Escudero JN, Youngquist BM, et al. CRISPR detection of circulating cell-free *Mycobacterium tuberculosis* DNA in adults and children, including children with HIV: a molecular diagnostics study. *The Lancet Microbe*. 2022;3(7):e482-e92.
  - ∘ Development of a CRISPR-based assay for the detection of cell-free M. tuberculosis DNA in serum, with test evaluations in two LMICs.
- Lee RA, Puig HD, Nguyen PQ, Angenent-Mari NM, Donghia NM, McGee JP, et al. Ultrasensitive CRISPR-based diagnostic for field-applicable detection of Plasmodium species in symptomatic and asymptomatic malaria. *Proceedings of the National Academy of Sciences*. 2020;117(41):25722–31.
  - ∘ Development of a CRISPR-based assay with simplified sample preparation to detect four major Plasmodium species.
- Lam MMC, Wick RR, Watts SC, Cerdeira LT, Wyres KL, Holt KE. A genomic surveillance framework and genotyping tool for *Klebsiella pneumoniae* and its related species complex. *Nature Communications*. 2021;12(1):4188.
  - ∘ A paper presenting the most comprehensive species-specific tools designed to identify clinically relevant traits, such as virulence and antibiotic resistance, using data from public database.
- Ma KC, Mortimer TD, Duckett MA, Hicks AL, Wheeler NE, Sánchez-Busó L, et al. Increased power from conditional bacterial genome-wide association identifies macrolide resistance mutations in *Neisseria gonorrhoeae*. *Nature Communications*. 2020;11(1):5374.
  - ∘ A methods paper highlighting an approach to detect genetic determinants linked to antibiotic resistance in a highly recombinogenic species like *Neisseria gonorrhoeae*.
- Farhat MR, Freschi L, Calderon R, Ioerger T, Snyder M, Meehan CJ, et al. GWAS for quantitative resistance phenotypes in *Mycobacterium tuberculosis* reveals resistance genes and regulatory regions. *Nature Communications*. 2019;10(1):2128.
  - ∘ A method paper describing a detection of genetic determinants linked to antibiotic resistance in a highly clonal species like *Mycobacterium tuberculosis*.

## Data Availability

No datasets were generated or analysed during the current study.

## References

[1] Ikuta KS, Swetschinski LR, Robles Aguilar G, Sharara F, Mestrovic T, Gray AP, et al. Global mortality associated with 33 bacterial pathogens in 2019: a systematic analysis for the Global Burden of Disease Study 2019. Lancet. 2022;400(10369):2221–48. 36423648 10.1016/S0140-6736(22)02185-7 PMC9763654

[2] Murray CJL, Ikuta KS, Sharara F, Swetschinski L, Robles Aguilar G, Gray A, et al. Global burden of bacterial antimicrobial resistance in 2019: a systematic analysis. Lancet. 2022;399(10325):629–55. 35065702 10.1016/S0140-6736(21)02724-0 PMC8841637

[3] Limmathurotsakul D, Golding N, Dance DAB, Messina JP, Pigott DM, Moyes CL, et al. Predicted global distribution of Burkholderia pseudomallei and burden of melioidosis. Nat Microbiol. 2016;1(1):15008. 27571754 10.1038/nmicrobiol.2015.8

[4] Organization WH. Global antimicrobial resistance and use surveillance system (GLASS) report 2022. Global antimicrobial resistance and use surveillance system (GLASS) report 2022 Geneva: World Health Organization; 2022 Licence: CC BY-NC-SA 30 IGO. 2022.

[5] Wilke M, Heinlein W, Stiefenhofer L, Bodmann KF. Clinical and economical improvements after introducing rapid identification of bacteria and early antibiotic susceptibility testing in sepsis and bloodstream infections. Results of the PHENOMENON study. GMS Infect Dis. 2020;8:Doc25. 33376664 10.3205/id000069 PMC7745702

[6] Uzuriaga M, Leiva J, Guillén-Grima F, Rua M, Yuste JR. Clinical impact of rapid bacterial microbiological Identification with the MALDI-TOF MS. Antibiotics. 2023;12(12):1660. 38136694 10.3390/antibiotics12121660 PMC10740418

[7] Eickelberg G, Sanchez-Pinto LN, Luo Y. Predictive modeling of bacterial infections and antibiotic therapy needs in critically ill adults. J Biomed Inform. 2020;109:103540. 32814200 10.1016/j.jbi.2020.103540 PMC7530142

[8] Giske CG, Turnidge J, Cantón R, Kahlmeter G. Update from the European Committee on Antimicrobial Susceptibility Testing (EUCAST). J Clin Microbiol. 2022;60(3):e0027621. 34346716 10.1128/jcm.00276-21 PMC8925892

[9] CLSI. Performance standards for antimicrobial disk susceptibility tests. 14th ed. CLSI standard M02. Clinical and Laboratory Standards Institute. 2024.

[10] Lagier JC, Edouard S, Pagnier I, Mediannikov O, Drancourt M, Raoult D. Current and past strategies for bacterial culture in clinical microbiology. Clin Microbiol Rev. 2015;28(1):208–36. 25567228 10.1128/CMR.00110-14 PMC4284306

[11] Ransom EM, Alipour Z, Wallace MA, Burnham CA. Evaluation of optimal blood culture incubation time to maximize clinically relevant results from a contemporary blood culture instrument and media system. J Clin Microbiol. 2021;59(3):e02459-20. 33239377 10.1128/JCM.02459-20 PMC8106720

[12] Quirino A, Marascio N, Peronace C, Gallo L, Barreca GS, Giancotti A, et al. Direct antimicrobial susceptibility testing (AST) from positive blood cultures using Microscan system for early detection of bacterial resistance phenotypes. Diagn Microbiol Infect Dis. 2021;101(2):115485. 34365091 10.1016/j.diagmicrobio.2021.115485

[13] Power RA, Parkhill J, de Oliveira T. Microbial genome-wide association studies: lessons from human GWAS. Nat Rev Genet. 2017;18(1):41–50. 27840430 10.1038/nrg.2016.132

[14] Narasimhan V, Kim H, Lee SH, Kang H, Siddique RH, Park H, et al. Nucleic Acid Amplification-Based Technologies (NAAT)—Toward accessible, autonomous, and mobile diagnostics. Adv Mater Technol. 2023;8(20):2300230.

[15] Miyakoshi A, Niimi H, Ueno T, Wakasugi M, Higashi Y, Miyajima Y, et al. Novel rapid method for identifying and quantifying pathogenic bacteria within four hours of blood collection. Sci Rep. 2024;14(1):1199. 38216600 10.1038/s41598-023-50864-0 PMC10786899

[16] Tan DHJ, Sun Q, Cheng X, Liu J, Liu J, Li Q, Dai L. Application of multiplex fluorescence polymerase chain reaction for detecting pathogenic bacteria in sputum samples from patients with lower respiratory tract infection. Infect Drug Resist. 2023;2023(16):6999–7005. 10.2147/IDR.S431425 PMC10625741 37933294

[17] Harris M, Fasolino T, Davis NJ, Ivankovic D, Brownlee N. Multiplex detection of antimicrobial resistance genes for rapid antibiotic guidance of urinary tract infections. Microbiol Res. 2023;14(2):591–602.

[18] Kalita MJ, Dutta K, Hazarika G, Dutta R, Kalita S, Das PP, et al. In-house reverse transcriptase polymerase chain reaction for detection of SARS-CoV-2 with increased sensitivity. Sci Rep. 2021;11(1):17878. 34504255 10.1038/s41598-021-97502-1 PMC8429455

[19] Sano H, Wakui A, Kawachi M, Washio J, Abiko Y, Mayanagi G, et al. Profiling system of oral microbiota utilizing polymerase chain reaction-restriction fragment length polymorphism analysis. J Oral Biosci. 2021;63(3):292–7. 34111508 10.1016/j.job.2021.05.003

[20] Hernández I, Sant C, Martínez R, Fernández C. Design of bacterial strain-specific qPCR assays using NGS data and publicly available resources and its application to track biocontrol strains. Front Microbiol. 2020;11:208. 32210925 10.3389/fmicb.2020.00208 PMC7077341

[21] Dung TTN, Phat VV, Vinh C, Lan NPH, Phuong NLN, Ngan LTQ, et al. Development and validation of multiplex real-time PCR for simultaneous detection of six bacterial pathogens causing lower respiratory tract infections and antimicrobial resistance genes. BMC Infect Dis. 2024;24(1):164. 38326753 10.1186/s12879-024-09028-2 PMC10848345

[22] Bang E, Oh S, Cho HW, Park D-h, Chang HE, Park JS, et al. Development of diagnostic tests for pathogen identification and detection of antimicrobial resistance on WHO global priority pathogens using modular real-time nucleic acid amplification test. Int Microbiol. 2023;26(3):563–77. 36646920 10.1007/s10123-023-00321-9

[23] Bang E, Oh S, Cho HW, Park DH, Chang HE, Park JS, et al. Development of diagnostic tests for pathogen identification and detection of antimicrobial resistance on WHO global priority pathogens using modular real-time nucleic acid amplification test. Int Microbiol. 2023;26(3):563–77. 36646920 10.1007/s10123-023-00321-9

[24] Li Y, Xiu L, Wang L, Zhang L, Wang F, Peng J. Rapid detection of antimicrobial resistance in Mycoplasma genitalium by high-resolution melting analysis with unlabeled probes. Microbiol Spectr. 2022;10(4):e01014-e1022. 35880894 10.1128/spectrum.01014-22 PMC9430336

[25] Pakdeerat S, Boonklang P, Angchagun K, Chomkatekaew C, Apichaidejudom N, Dokket Y, et al. Benchmarking CRISPR-BP34 for point-of-care melioidosis detection in low-income and middle-income countries: a molecular diagnostics study. Lancet Microbe. 2024;5(4):e379–89. 38493790 10.1016/S2666-5247(23)00378-6 PMC10990966

[26] Trung NT, Son LHP, Hien TX, Quyen DT, Bang MH, Song LH. CRISPR-Cas12a combination to alleviate the false-positive in loop-mediated isothermal amplification-based diagnosis of Neisseria meningitidis. BMC Infect Dis. 2022;22(1):429. 35508977 10.1186/s12879-022-07363-w PMC9066958

[27] Kham-Kjing N, Ngo-Giang-Huong N, Tragoolpua K, Khamduang W, Hongjaisee S. Highly specific and rapid detection of hepatitis c virus using RT-LAMP-Coupled CRISPR-Cas12 assay. Diagnostics (Basel). 2022;12(7):1524. 35885430 10.3390/diagnostics12071524 PMC9317538

[28] Thakku SG, Lirette J, Murugesan K, Chen J, Theron G, Banaei N, et al. Genome-wide tiled detection of circulating Mycobacterium tuberculosis cell-free DNA using Cas13. Nat Commun. 2023;14(1):1803. 37002219 10.1038/s41467-023-37183-8 PMC10064635

[29] Tomita N, Mori Y, Kanda H, Notomi T. Loop-mediated isothermal amplification (LAMP) of gene sequences and simple visual detection of products. Nat Protoc. 2008;3(5):877–82. 18451795 10.1038/nprot.2008.57

[30] Piepenburg O, Williams CH, Stemple DL, Armes NA. DNA Detection Using Recombination Proteins. PLoS Biol. 2006;4(7):e204. 16756388 10.1371/journal.pbio.0040204 PMC1475771

[31] Park J-W. Principles and applications of loop-mediated isothermal amplification to point-of-care tests. Biosensors. 2022;12:857. 36290994 10.3390/bios12100857 PMC9599884

[32] Srivastava P, Prasad D. Isothermal nucleic acid amplification and its uses in modern diagnostic technologies. 3 Biotech. 2023;13(6):200. 37215369 10.1007/s13205-023-03628-6 PMC10193355

[33] Oliveira BB, Veigas B, Baptista PV. Isothermal amplification of nucleic acids: the race for the next “gold standard.” Front Sens. 2021;2:752600.

[34] Hsieh K, Mage PL, Csordas AT, Eisenstein M, Tom SH. Simultaneous elimination of carryover contamination and detection of DNA with uracil-DNA-glycosylase-supplemented loop-mediated isothermal amplification (UDG-LAMP). Chem Commun. 2014;50(28):3747–9. 10.1039/c4cc00540f 24577617

[35] Paik I, Ngo PHT, Shroff R, Diaz DJ, Maranhao AC, Walker DJF, et al. Improved Bst DNA polymerase variants derived via a machine learning approach. Biochemistry. 2023;62(2):410–8. 34762799 10.1021/acs.biochem.1c00451 PMC9514386

[36] Dangerfield TL, Paik I, Bhadra S, Johnson KA, Ellington AD. Kinetics of elementary steps in loop-mediated isothermal amplification (LAMP) show that strand invasion during initiation is rate-limiting. Nucleic Acids Res. 2022;51(1):488–99. 10.1093/nar/gkac1221 PMC9841402 36583345

[37] Li J, Macdonald J, von Stetten F. Review: a comprehensive summary of a decade development of the recombinase polymerase amplification. Analyst. 2019;144(1):31–67. 10.1039/c8an01621f 30426974

[38] Ae LU. Computer program for primer design for loop-mediated isothermal amplification (LAMP). Adv Eng Res (Rostov-on-Don). 2024;24(1):98–108.

[39] Higgins M, Ravenhall M, Ward D, Phelan J, Ibrahim A, Forrest MS, et al. PrimedRPA: primer design for recombinase polymerase amplification assays. Bioinformatics. 2018;35(4):682–4. 10.1093/bioinformatics/bty701 PMC6379019 30101342

[40] Gavrilov M, Yang JYC, Zou RS, Ma W, Lee C-Y, Mohapatra S, et al. Engineered helicase replaces thermocycler in DNA amplification while retaining desired PCR characteristics. Nat Commun. 2022;13(1):6312. 36274095 10.1038/s41467-022-34076-0 PMC9588791

[41] Garafutdinov RR, Sakhabutdinova AR, Gilvanov AR, Chemeris AV. Rolling circle amplification as a universal method for the analysis of a wide range of biological targets. Russ J Bioorg Chem. 2021;47(6):1172–89. 34931113 10.1134/S1068162021060078 PMC8675116

[42] Qian J, Ferguson TM, Shinde DN, Ramírez-Borrero AJ, Hintze A, Adami C, et al. Sequence dependence of isothermal DNA amplification via EXPAR. Nucleic Acids Res. 2012;40(11):e87-e. 22416064 10.1093/nar/gks230 PMC3367216

[43] Walker GT, Fraiser MS, Schram JL, Little MC, Nadeau JG, Malinowski DP. Strand displacement amplification–an isothermal, in vitro DNA amplification technique. Nucleic Acids Res. 1992;20(7):1691–6. 1579461 10.1093/nar/20.7.1691 PMC312258

[44] Hoser MJ, Mansukoski HK, Morrical SW, Eboigbodin KE. Strand Invasion Based Amplification (SIBA®): a novel isothermal DNA amplification technology demonstrating high specificity and sensitivity for a single molecule of target analyte. PLoS One. 2014;9(11):e112656. 25419812 10.1371/journal.pone.0112656 PMC4242538

[45] Compton J. Nucleic acid sequence-based amplification. Nature. 1991;350(6313):91–2. 1706072 10.1038/350091a0

[46] Xu G, Hu L, Zhong H, Wang H, Yusa S, Weiss TC, et al. Cross priming amplification: mechanism and optimization for isothermal DNA amplification. Sci Rep. 2012;2:246. 22355758 10.1038/srep00246 PMC3271364

[47] Mann JG, Pitts RJ. PrimedSherlock: a tool for rapid design of highly specific CRISPR-Cas12 crRNAs. BMC Bioinformatics. 2022;23(1):428. 36241974 10.1186/s12859-022-04968-5 PMC9569017

[48] Labun K, Krause M, Torres Cleuren Y, Valen E. CRISPR genome editing made easy through the CHOPCHOP website. Curr Protoc. 2021;1(4):e46. 33905612 10.1002/cpz1.46

[49] Naito Y, Hino K, Bono H, Ui-Tei K. CRISPRdirect: software for designing CRISPR/Cas guide RNA with reduced off-target sites. Bioinformatics. 2015;31(7):1120–3. 25414360 10.1093/bioinformatics/btu743 PMC4382898

[50] Asadbeigi A, Norouzi M, Vafaei Sadi MS, Saffari M, Bakhtiarizadeh MR. CaSilico: a versatile CRISPR package for in silico CRISPR RNA designing for Cas12, Cas13, and Cas14. Front Bioeng Biotechnol. 2022;10:957131. 36017348 10.3389/fbioe.2022.957131 PMC9395711

[51] Chuai G, Ma H, Yan J, Chen M, Hong N, Xue D, et al. DeepCRISPR: optimized CRISPR guide RNA design by deep learning. Genome Biol. 2018;19(1):80. 29945655 10.1186/s13059-018-1459-4 PMC6020378

[52] Poudel R, Rodriguez LT, Reisch CR, Rivers AR. GuideMaker: Software to design CRISPR-Cas guide RNA pools in non-model genomes. GigaScience. 2022;11:giac007. 10.1093/gigascience/giac007 PMC8975720 35365834

[53] Moreno-Mateos MA, Vejnar CE, Beaudoin JD, Fernandez JP, Mis EK, Khokha MK, et al. CRISPRscan: designing highly efficient sgRNAs for CRISPR-Cas9 targeting in vivo. Nat Methods. 2015;12(10):982–8. 26322839 10.1038/nmeth.3543 PMC4589495

[54] Zhu H, Liang C. CRISPR-DT: designing gRNAs for the CRISPR-Cpf1 system with improved target efficiency and specificity. Bioinformatics. 2019;35(16):2783–9. 30615056 10.1093/bioinformatics/bty1061

[55] Benchling [Biology Software]. 2024. Retrieved from https://benchling.com

[56] Low SJ, O’Neill M, Kerry WJ, Wild N, Krysiak M, Nong Y, et al. PathoGD: an integrative genomics approach for CRISPR-based target design of rapid pathogen diagnostics. bioRxiv. 10.1101/2024.05.14.593882

[57] Chen JS, Ma E, Harrington LB, Da Costa M, Tian X, Palefsky JM, et al. CRISPR-Cas12a target binding unleashes indiscriminate single-stranded DNase activity. Science. 2018;360(6387):436–9. 29449511 10.1126/science.aar6245 PMC6628903

[58] Gootenberg JS, Abudayyeh OO, Lee JW, Essletzbichler P, Dy AJ, Joung J, et al. Nucleic acid detection with CRISPR-Cas13a/C2c2. Science. 2017;356(6336):438–42. 28408723 10.1126/science.aam9321 PMC5526198

[59] Collins DM, Stephens DM. Identification of an insertion sequence, IS1081, in Mycobacterium bovis. FEMS Microbiol Lett. 1991;83(1):11–5. 10.1016/0378-1097(91)90435-d 1663885

[60] Comín J, Otal I, Samper S. In-depth analysis of IS6110 genomic variability in the mycobacterium tuberculosis complex. Front Microbiol. 2022;13:767912. 35283840 10.3389/fmicb.2022.767912 PMC8912993

[61] Jacobsen T, Ttofali F, Liao C, Manchalu S, Gray BN, Beisel CL. Characterization of Cas12a nucleases reveals diverse PAM profiles between closely-related orthologs. Nucleic Acids Res. 2020;48(10):5624–38. 32329776 10.1093/nar/gkaa272 PMC7261169

[62] Collias D, Beisel CL. CRISPR technologies and the search for the PAM-free nuclease. Nat Commun. 2021;12(1):555. 33483498 10.1038/s41467-020-20633-y PMC7822910

[63] Harrington LB, Burstein D, Chen JS, Paez-Espino D, Ma E, Witte IP, et al. Programmed DNA destruction by miniature CRISPR-Cas14 enzymes. Science. 2018;362(6416):839–42. 30337455 10.1126/science.aav4294 PMC6659742

[64] Zhang H-X, Zhang C, Lu S, Tong X, Zhang K, Yin H, et al. Cas12a-based one-pot SNP detection with high accuracy. Cell Insight. 2023;2(2):100080. 37193068 10.1016/j.cellin.2023.100080 PMC10134196

[65] Kim H, Lee W-j, Oh Y, Kang S-H, Hur JK, Lee H, et al. Enhancement of target specificity of CRISPR–Cas12a by using a chimeric DNA–RNA guide. Nucleic Acids Res. 2020;48(15):8601–16. 32687187 10.1093/nar/gkaa605 PMC7470973

[66] Nguyen LT, Rananaware SR, Yang LG, Macaluso NC, Ocana-Ortiz JE, Meister KS, et al. Engineering highly thermostable Cas12b via de novo structural analyses for one-pot detection of nucleic acids. Cell Rep Med. 2023;4(5):101037. 37160120 10.1016/j.xcrm.2023.101037 PMC10213852

[67] Nguyen LT, Macaluso NC, Pizzano BLM, Cash MN, Spacek J, Karasek J, et al. A thermostable Cas12b from Brevibacillus leverages one-pot discrimination of SARS-CoV-2 variants of concern. EBioMedicine. 2022;77:103926. 35290826 10.1016/j.ebiom.2022.103926 PMC8917962

[68] Nalefski EA, Patel N, Leung PJY, Islam Z, Kooistra RM, Parikh I, et al. Kinetic analysis of Cas12a and Cas13a RNA-Guided nucleases for development of improved CRISPR-Based diagnostics. iScience. 2021;24(9):102996. 34505008 10.1016/j.isci.2021.102996 PMC8411246

[69] Emery NJ, Majumder S, Liu AP. Synergistic and non-specific nucleic acid production by T7 RNA polymerase and Bsu DNA polymerase catalyzed by single-stranded polynucleotides. Synth Syst Biotechnol. 2018;3(2):130–4. 29900426 10.1016/j.synbio.2018.02.005 PMC5995454

[70] Yang J, Song Y, Deng X, Vanegas JA, You Z, Zhang Y, et al. Engineered LwaCas13a with enhanced collateral activity for nucleic acid detection. Nat Chem Biol. 2023;19(1):45–54. 36138140 10.1038/s41589-022-01135-y

[71] Gootenberg JS, Abudayyeh OO, Kellner MJ, Joung J, Collins JJ, Zhang F. Multiplexed and portable nucleic acid detection platform with Cas13, Cas12a, and Csm6. Science. 2018;360(6387):439–44. 29449508 10.1126/science.aaq0179 PMC5961727

[72] Mahas A, Marsic T, Lopez-Portillo Masson M, Wang Q, Aman R, Zheng C, et al. Characterization of a thermostable Cas13 enzyme for one-pot detection of SARS-CoV-2. Proc Natl Acad Sci. 2022;119(28):e2118260119. 35763567 10.1073/pnas.2118260119 PMC9282225

[73] Huang Z, LaCourse SM, Kay AW, Stern J, Escudero JN, Youngquist BM, et al. CRISPR detection of circulating cell-free <em>Mycobacterium tuberculosis</em> DNA in adults and children, including children with HIV: a molecular diagnostics study. Lancet Microbe. 2022;3(7):e482–92. 35659882 10.1016/S2666-5247(22)00087-8 PMC9300929

[74] Li H, Cui X, Sun L, Deng X, Liu S, Zou X, et al. High concentration of Cas12a effector tolerates more mismatches on ssDNA. FASEB J. 2021;35(1):e21153. 33159392 10.1096/fj.202001475R

[75] Liu Y, Liu H, Yu G, Sun W, Aizaz M, Yang G, et al. One-tube RPA-CRISPR Cas12a/Cas13a rapid detection of methicillin-resistant Staphylococcus aureus. Anal Chim Acta. 2023;1278:341757. 37709482 10.1016/j.aca.2023.341757

[76] Cao Y, Tian Y, Huang J, Xu L, Fan Z, Pan Z, et al. CRISPR/Cas13-assisted carbapenem-resistant Klebsiella pneumoniae detection. J Microbiol Immunol Infect. 2024;57(1):118–27. 37963801 10.1016/j.jmii.2023.10.010

[77] Liang M, Xiao B, Chen L, Huang X, Li J, Kuang Z, et al. Rapid detection of blaKPC in carbapenem-resistant Enterobacterales based on CRISPR/Cas13a. Curr Microbiol. 2023;80(11):352. 37737960 10.1007/s00284-023-03457-z PMC10638124

[78] Zhou Z, Liang L, Liao C, Pan L, Wang C, Ma J, et al. A multiplex RPA coupled with CRISPR-Cas12a system for rapid and cost-effective identification of carbapenem-resistant Acinetobacter baumannii. Front Microbiol. 2024;15:1359976. 38516017 10.3389/fmicb.2024.1359976 PMC10956356

[79] Luo H, Zeng L, Yin X, Pan Y, Yang J, Liu M, et al. An isothermal CRISPR-based diagnostic assay for Neisseria gonorrhoeae and Chlamydia trachomatis detection. Microbiol Spectr. 2023;11(6):e0046423. 37882532 10.1128/spectrum.00464-23 PMC10715037

[80] Wang Y, Liang X, Jiang Y, Dong D, Zhang C, Song T, et al. Novel fast pathogen diagnosis method for severe pneumonia patients in the intensive care unit: randomized clinical trial. eLife. 2022;11:e79014. 36205312 10.7554/eLife.79014 PMC9605691

[81] Liu S, Huang S, Li F, Sun Y, Fu J, Xiao F, et al. Rapid detection of Pseudomonas aeruginosa by recombinase polymerase amplification combined with CRISPR-Cas12a biosensing system. Front Cell Infect Microbiol. 2023;13:1239269. 37637458 10.3389/fcimb.2023.1239269 PMC10449609

[82] Wang S, Wang S, Hao T, Zhu S, Qiu X, Li Y, et al. Detection of Salmonella DNA and drug-resistance mutation by PCR-based CRISPR-lbCas12a system. AMB Express. 2023;13(1):100. 37750967 10.1186/s13568-023-01588-x PMC10522547

[83] Shi Y, Kang L, Mu R, Xu M, Duan X, Li Y, et al. CRISPR/Cas12a-enhanced loop-mediated isothermal amplification for the visual detection of Shigella flexneri. Front Bioeng Biotechnol. 2022;10:845688. 35265606 10.3389/fbioe.2022.845688 PMC8899461

[84] Tan M, Liao C, Liang L, Yi X, Zhou Z, Wei G. Recent advances in recombinase polymerase amplification: Principle, advantages, disadvantages and applications. Front Cell Infect Microbiol. 2022;12:1019071. 36519130 10.3389/fcimb.2022.1019071 PMC9742450

[85] Özay B, McCalla SE. A review of reaction enhancement strategies for isothermal nucleic acid amplification reactions. Sensors Actuators Rep. 2021;3:100033.

[86] Swarts DC, van der Oost J, Jinek M. Structural basis for guide RNA processing and seed-dependent DNA targeting by CRISPR-Cas12a. Mol Cell. 2017;66(2):221-33.e4. 28431230 10.1016/j.molcel.2017.03.016 PMC6879319

[87] Safari F, Zare K, Negahdaripour M, Barekati-Mowahed M, Ghasemi Y. CRISPR Cpf1 proteins: structure, function and implications for genome editing. Cell Biosci. 2019;9(1):36. 31086658 10.1186/s13578-019-0298-7 PMC6507119

[88] Paul B, Montoya G. CRISPR-Cas12a: Functional overview and applications. Biomed J. 2020;43(1):8–17. 32200959 10.1016/j.bj.2019.10.005 PMC7090318

[89] Abudayyeh OO, Gootenberg JS, Essletzbichler P, Han S, Joung J, Belanto JJ, et al. RNA targeting with CRISPR–Cas13. Nature. 2017;550(7675):280–4. 28976959 10.1038/nature24049 PMC5706658

[90] Freschi L, Vargas R Jr, Husain A, Kamal SMM, Skrahina A, Tahseen S, et al. Population structure, biogeography and transmissibility of Mycobacterium tuberculosis. Nat Commun. 2021;12(1):6099. 34671035 10.1038/s41467-021-26248-1 PMC8528816

[91] Croucher NJ, Harris SR, Fraser C, Quail MA, Burton J, van der Linden M, et al. Rapid pneumococcal evolution in response to clinical interventions. Science. 2011;331(6016):430–4. 21273480 10.1126/science.1198545 PMC3648787

[92] Chewapreecha C, Harris SR, Croucher NJ, Turner C, Marttinen P, Cheng L, et al. Dense genomic sampling identifies highways of pneumococcal recombination. Nat Genet. 2014;46(3):305–9. 24509479 10.1038/ng.2895 PMC3970364

[93] Nandi T, Holden MT, Didelot X, Mehershahi K, Boddey JA, Beacham I, et al. Burkholderia pseudomallei sequencing identifies genomic clades with distinct recombination, accessory, and epigenetic profiles. Genome Res. 2015;25(1):129–41. 25236617 10.1101/gr.177543.114 PMC4317168

[94] De Silva D, Peters J, Cole K, Cole MJ, Cresswell F, Dean G, et al. Whole-genome sequencing to determine transmission of Neisseria gonorrhoeae: an observational study. Lancet Infect Dis. 2016;16(11):1295–303. 27427203 10.1016/S1473-3099(16)30157-8 PMC5086424

[95] Torrance EL, Burton C, Diop A, Bobay L-M. Evolution of homologous recombination rates across bacteria. Proc Natl Acad Sci. 2024;121(18):e2316302121. 38657048 10.1073/pnas.2316302121 PMC11067023

[96] Lam MMC, Wick RR, Watts SC, Cerdeira LT, Wyres KL, Holt KE. A genomic surveillance framework and genotyping tool for Klebsiella pneumoniae and its related species complex. Nat Commun. 2021;12(1):4188. 34234121 10.1038/s41467-021-24448-3 PMC8263825

[97] Hanage WP, Fraser C, Spratt BG. Fuzzy species among recombinogenic bacteria. BMC Biol. 2005;3:6. 15752428 10.1186/1741-7007-3-6 PMC554772

[98] Harvey WT, Carabelli AM, Jackson B, Gupta RK, Thomson EC, Harrison EM, et al. SARS-CoV-2 variants, spike mutations and immune escape. Nat Rev Microbiol. 2021;19(7):409–24. 34075212 10.1038/s41579-021-00573-0 PMC8167834

[99] Chaguza C, Senghore M, Bojang E, Gladstone RA, Lo SW, Tientcheu PE, et al. Within-host microevolution of Streptococcus pneumoniae is rapid and adaptive during natural colonisation. Nat Commun. 2020;11(1):3442. 32651390 10.1038/s41467-020-17327-w PMC7351774

[100] McLeod DV, Gandon S. Effects of epistasis and recombination between vaccine-escape and virulence alleles on the dynamics of pathogen adaptation. Nat Ecol Evol. 2022;6(6):786–93. 35437006 10.1038/s41559-022-01709-y

[101] Blackwell GA, Hunt M, Malone KM, Lima L, Horesh G, Alako BTF, et al. Exploring bacterial diversity via a curated and searchable snapshot of archived DNA sequences. PLoS Biol. 2021;19(11):e3001421. 34752446 10.1371/journal.pbio.3001421 PMC8577725

[102] Tonkin-Hill G, MacAlasdair N, Ruis C, Weimann A, Horesh G, Lees JA, et al. Producing polished prokaryotic pangenomes with the Panaroo pipeline. Genome Biol. 2020;21(1):180. 32698896 10.1186/s13059-020-02090-4 PMC7376924

[103] Figueroa JL, III, Redinbo A, Panyala A, Colby S, Friesen ML, Tiemann L, et al. MerCat2: a versatile k-mer counter and diversity estimator for database-independent property analysis obtained from omics data. Bioinform Adv. 2024;4(1):vbae061. 10.1093/bioadv/vbae061 PMC11090762 38745763

[104] Moeckel C, Mareboina M, Konnaris MA, Chan CSY, Mouratidis I, Montgomery A, et al. A survey of k-mer methods and applications in bioinformatics. Comput Struct Biotechnol J. 2024;23:2289–303. 38840832 10.1016/j.csbj.2024.05.025 PMC11152613

[105] Kapli P, Yang Z, Telford MJ. Phylogenetic tree building in the genomic age. Nat Rev Genet. 2020;21(7):428–44. 32424311 10.1038/s41576-020-0233-0

[106] O'Toole Á, Scher E, Underwood A, Jackson B, Hill V, McCrone JT, et al. Assignment of epidemiological lineages in an emerging pandemic using the pangolin tool. Virus Evol. 2021;7(2):veab064. 10.1093/ve/veab064 PMC8344591 34527285

[107] Mosquera-Rendón J, Moreno-Herrera CX, Robledo J, Hurtado-Páez U. Genome-Wide Association Studies (GWAS) approaches for the detection of genetic variants associated with antibiotic resistance: a systematic review. Microorganisms. 2023;11(12):2866. 38138010 10.3390/microorganisms11122866 PMC10745584

[108] Allen JP, Snitkin E, Pincus NB, Hauser AR. Forest and trees: exploring bacterial virulence with genome-wide association studies and machine learning. Trends Microbiol. 2021;29(7):621–33. 33455849 10.1016/j.tim.2020.12.002 PMC8187264

[109] Naz S, Paritosh K, Sanyal P, Khan S, Singh Y, Varshney U, et al. GWAS and functional studies suggest a role for altered DNA repair in the evolution of drug resistance in Mycobacterium tuberculosis. Elife. 2023;12:e75860. 36695572 10.7554/eLife.75860 PMC9876569

[110] Farhat MR, Freschi L, Calderon R, Ioerger T, Snyder M, Meehan CJ, et al. GWAS for quantitative resistance phenotypes in Mycobacterium tuberculosis reveals resistance genes and regulatory regions. Nat Commun. 2019;10(1):2128. 31086182 10.1038/s41467-019-10110-6 PMC6513847

[111] Chewapreecha C, Marttinen P, Croucher NJ, Salter SJ, Harris SR, Mather AE, et al. Comprehensive identification of single nucleotide polymorphisms associated with beta-lactam resistance within pneumococcal mosaic genes. PLoS Genet. 2014;10(8):e1004547. 25101644 10.1371/journal.pgen.1004547 PMC4125147

[112] Spadar A, Perdigão J, Campino S, Clark TG. Genomic analysis of hypervirulent Klebsiella pneumoniae reveals potential genetic markers for differentiation from classical strains. Sci Rep. 2022;12(1):13671. 35953553 10.1038/s41598-022-17995-2 PMC9372168

[113] Pei N, Sun W, He J, Li Y, Chen X, Liang T, et al. Genome-wide association study of Klebsiella pneumoniae identifies variations linked to carbapenems resistance. Front Microbiol. 2022;13:997769. 36386631 10.3389/fmicb.2022.997769 PMC9664935

[114] Earle SG, Lobanovska M, Lavender H, Tang C, Exley RM, Ramos-Sevillano E, et al. Genome-wide association studies reveal the role of polymorphisms affecting factor H binding protein expression in host invasion by Neisseria meningitidis. PLoS Pathog. 2021;17(10):e1009992. 34662348 10.1371/journal.ppat.1009992 PMC8553145

[115] Golparian D, Cole MJ, Sánchez-Busó L, Day M, Jacobsson S, Uthayakumaran T, et al. Antimicrobial-resistant Neisseria gonorrhoeae in Europe in 2020 compared with in 2013 and 2018: a retrospective genomic surveillance study. Lancet Microbe. 2024;5(5):e478–88. 38614111 10.1016/S2666-5247(23)00370-1

[116] Ma KC, Mortimer TD, Duckett MA, Hicks AL, Wheeler NE, Sánchez-Busó L, et al. Increased power from conditional bacterial genome-wide association identifies macrolide resistance mutations in Neisseria gonorrhoeae. Nat Commun. 2020;11(1):5374. 33097713 10.1038/s41467-020-19250-6 PMC7584619

[117] Chewapreecha C, Mather AE, Harris SR, Hunt M, Holden MTG, Chaichana C, et al. Genetic variation associated with infection and the environment in the accidental pathogen Burkholderia pseudomallei. Commun Biol. 2019;2:428. 31799430 10.1038/s42003-019-0678-x PMC6874650

[118] Murugan K, Seetharam AS, Severin AJ, Sashital DG. CRISPR-Cas12a has widespread off-target and dsDNA-nicking effects. J Biol Chem. 2020;295(17):5538–53. 32161115 10.1074/jbc.RA120.012933 PMC7186167

[119] Wessels HH, Méndez-Mancilla A, Guo X, Legut M, Daniloski Z, Sanjana NE. Massively parallel Cas13 screens reveal principles for guide RNA design. Nat Biotechnol. 2020;38(6):722–7. 32518401 10.1038/s41587-020-0456-9 PMC7294996

[120] Arredondo-Alonso S, Blundell-Hunter G, Fu Z, Gladstone RA, Fillol-Salom A, Loraine J, et al. Evolutionary and functional history of the Escherichia coli K1 capsule. Nat Commun. 2023;14(1):3294. 37322051 10.1038/s41467-023-39052-w PMC10272209

[121] Aldawood E, Roberts IS. Regulation of Escherichia coli Group 2 capsule gene expression: a mini review and update. Front Microbiol. 2022;13:858767. 35359738 10.3389/fmicb.2022.858767 PMC8960920

[122] Meumann EM, Limmathurotsakul D, Dunachie SJ, Wiersinga WJ, Currie BJ. Burkholderia pseudomallei and melioidosis. Nat Rev Microbiol. 2024;22(3):155–69. 37794173 10.1038/s41579-023-00972-5

[123] Burnard D, Bauer MJ, Falconer C, Gassiep I, Norton RE, Paterson DL, et al. Clinical Burkholderia pseudomallei isolates from north Queensland carry diverse bimABm genes that are associated with central nervous system disease and are phylogenomically distinct from other Australian strains. PLoS Negl Trop Dis. 2022;16(6):e0009482. 35700198 10.1371/journal.pntd.0009482 PMC9236262

[124] Mohapatra PR, Mishra B. Burden of melioidosis in India and South Asia: Challenges and ways forward. Lancet Reg Health Southeast Asia. 2022;2:100004. 37383295 10.1016/j.lansea.2022.03.004 PMC10306050

[125] Currie BJ, Mayo M, Ward LM, Kaestli M, Meumann EM, Webb JR, et al. The Darwin Prospective Melioidosis Study: a 30-year prospective, observational investigation. Lancet Infect Dis. 2021;21(12):1737–46. 34303419 10.1016/S1473-3099(21)00022-0

[126] Selvam K, Ganapathy T, Najib MA, Khalid MF, Abdullah NA, Harun A, et al. Burden and risk factors of melioidosis in Southeast Asia: a scoping review. Int J Environ Res Public Health. 2022;19(23). 10.3390/ijerph192315475 PMC9741171 36497549

[127] Anggraini D, Siregar FM, Rosdiana D, Kemal RA, Yovi I, Triani ZD, et al. Epidemiology and genetic diversity of Burkholderia pseudomallei from Riau province, Indonesia. PLoS Negl Trop Dis. 2024;18(5):e0012195. 38805481 10.1371/journal.pntd.0012195 PMC11161056

[128] Swe MMM, Win MM, Cohen J, Phyo AP, Lin HN, Soe K, et al. Geographical distribution of Burkholderia pseudomallei in soil in Myanmar. PLoS Negl Trop Dis. 2021;15(5):e0009372. 34029325 10.1371/journal.pntd.0009372 PMC8143414

[129] Chantratita N, Phunpang R, Yarasai A, Dulsuk A, Yimthin T, Onofrey LA, et al. Characteristics and one year outcomes of melioidosis patients in Northeastern Thailand: A prospective, multicenter cohort study. Lancet Reg Health Southeast Asia. 2023;9. 10.1016/j.lansea.2022.100118 PMC9788505 36570973

[130] Seng R, Chomkatekaew C, Tandhavanant S, Saiprom N, Phunpang R, Thaipadungpanit J, et al. Genetic diversity, determinants, and dissemination of Burkholderia pseudomallei lineages implicated in melioidosis in Northeast Thailand. Nat Commun. 2024;15(1):5699. 38972886 10.1038/s41467-024-50067-9 PMC11228029

[131] Wuthiekanun V, Chierakul W, Rattanalertnavee J, Langa S, Sirodom D, Wattanawaitunechai C, et al. Serological evidence for increased human exposure to Burkholderia pseudomallei following the tsunami in southern Thailand. J Clin Microbiol. 2006;44(1):239–40. 16390980 10.1128/JCM.44.1.239-240.2006 PMC1351951

[132] Lee Y-M, Park HJ. Clinical characteristics and predictors of mortality in patients with burkholderia cepacia complex bactremia. Open Forum Infect Dis. 2015;2(suppl_1).

[133] Wiersinga WJ, Virk HS, Torres AG, Currie BJ, Peacock SJ, Dance DAB, et al. Melioidosis. Nat Rev Dis Primers. 2018;4:17107. 29388572 10.1038/nrdp.2017.107 PMC6456913

[134] Lau SK, Sridhar S, Ho CC, Chow WN, Lee KC, Lam CW, et al. Laboratory diagnosis of melioidosis: past, present and future. Exp Biol Med (Maywood). 2015;240(6):742–51. 25908634 10.1177/1535370215583801 PMC4935216

[135] Furin J, Cox H, Pai M. Tuberculosis. Lancet. 2019;393(10181):1642–56. 30904262 10.1016/S0140-6736(19)30308-3

[136] Arezzo F, Cazzato G, Loizzi V, Ingravallo G, Resta L, Cormio G. Peritoneal tuberculosis mimicking ovarian cancer: gynecologic ultrasound evaluation with histopathological confirmation. Gastroenterol Insights. 2021;12(2):278–82.

[137] Hang T-X, Fang G, Huang Y, Hu C-M, Chen W. Misdiagnosis of a multi-organ involvement hematogenous disseminated tuberculosis as metastasis: a case report and literature review. Infect Dis Poverty. 2020;9(1):66. 32517798 10.1186/s40249-020-00681-8 PMC7285721

[138] Kim C, Ko Y, Moon JW, Park YB, Park SY, Ban GY, et al. Incidence, risk factors, and final causes for misdiagnosis of tuberculosis in the Republic of Korea: a population-based longitudinal analysis. Eur Respir J. 2022;60(4). 10.1183/13993003.01461-2022 36104288

[139] Xiang Y, Huang C, He Y, Zhang Q. Cancer or Tuberculosis: A Comprehensive Review of the Clinical and Imaging Features in Diagnosis of the Confusing Mass. Front Oncol. 2021;11:644150. 33996560 10.3389/fonc.2021.644150 PMC8113854

[140] Campelo TA, Cardoso de Sousa PR, Nogueira LL, Frota CC, Zuquim Antas PR. Revisiting the methods for detecting Mycobacterium tuberculosis: what has the new millennium brought thus far? Access Microbiol 2021;3(8):000245 10.1099/acmi.0.000245 PMC8479963 34595396

[141] Elbrolosy AM, El Helbawy RH, Mansour OM, Latif RA. Diagnostic utility of GeneXpert MTB/RIF assay versus conventional methods for diagnosis of pulmonary and extra-pulmonary tuberculosis. BMC Microbiol. 2021;21(1):144. 33980173 10.1186/s12866-021-02210-5 PMC8117635

[142] Borodulina EA, Piskun VV, Uraksina MV, Shubina AT. Molecular genetic tests GeneXpert MTB/RIF and Xpert MTB/RIF (Ultra) in the diagnosis of tuberculosis (review of literature). Klin Lab Diagn. 2022;67(9):544–9. 36099465 10.51620/0869-2084-2022-67-9-544-549

[143] Kay AW, Ness T, Verkuijl SE, Viney K, Brands A, Masini T, et al. Xpert MTB/RIF Ultra assay for tuberculosis disease and rifampicin resistance in children. Cochrane Database Syst Rev. 2022(9). 10.1002/14651858.CD013359.pub3 PMC9446385 36065889

[144] Arora J, Suresh N, Porwal C, Pandey P, Pande JN, Singh UB. Genotyping Mycobacterium tuberculosis isolates with few copies of IS6110: Value of additional genetic markers. Infect Genet Evol. 2020;81:104230. 10.1016/j.meegid.2020.104230. 32035976 10.1016/j.meegid.2020.104230

[145] Land KJ, Boeras DI, Chen X-S, Ramsay AR, Peeling RW. REASSURED diagnostics to inform disease control strategies, strengthen health systems and improve patient outcomes. Nat Microbiol. 2019;4(1):46–54. 30546093 10.1038/s41564-018-0295-3 PMC7097043

[146] Myhrvold C, Freije CA, Gootenberg JS, Abudayyeh OO, Metsky HC, Durbin AF, et al. Field-deployable viral diagnostics using CRISPR-Cas13. Science. 2018;360(6387):444–8. 29700266 10.1126/science.aas8836 PMC6197056

[147] Lee RA, Puig H, Nguyen PQ, Angenent-Mari NM, Donghia NM, McGee JP, et al. Ultrasensitive CRISPR-based diagnostic for field-applicable detection of Plasmodium species in symptomatic and asymptomatic malaria. Proc Natl Acad Sci U S A. 2020;117(41):25722–31. 32958655 10.1073/pnas.2010196117 PMC7568265

[148] Patchsung M, Jantarug K, Pattama A, Aphicho K, Suraritdechachai S, Meesawat P, et al. Clinical validation of a Cas13-based assay for the detection of SARS-CoV-2 RNA. Nat Biomed Eng. 2020;4(12):1140–9. 32848209 10.1038/s41551-020-00603-x

[149] Magnetic GA, Isolation B-B. In: Gautam A, editor. DNA and RNA Isolation Techniques for Non-Experts. Cham: Springer International Publishing; 2022. p. 111–7.

[150] Qian S, Chen Y, Peng C, Wang X, Wu H, Che Y, et al. Dipstick-based rapid nucleic acids purification and CRISPR/Cas12a-mediated isothermal amplification for visual detection of African swine fever virus. Talanta. 2022;242:123294. 35149424 10.1016/j.talanta.2022.123294

[151] Mason MG, Botella JR. Rapid (30-second), equipment-free purification of nucleic acids using easy-to-make dipsticks. Nat Protoc. 2020;15(11):3663–77. 33005038 10.1038/s41596-020-0392-7 PMC7528719

[152] Obino D, Vassalli M, Franceschi A, Alessandrini A, Facci P, Viti F. An overview on microfluidic systems for nucleic acids extraction from human raw samples. Sensors. 2021;21(9):3058. 33925730 10.3390/s21093058 PMC8125272

[153] Avaro AS, Santiago JG. A critical review of microfluidic systems for CRISPR assays. Lab Chip. 2023;23(5):938–63. 36601854 10.1039/d2lc00852a

[154] Qiu X, Liu X, Ma X, Wang R, Chen S, Li F, et al. One-pot isothermal LAMP-CRISPR-based assay for Klebsiella pneumoniae detection. Microbiol Spectr. 2022;10(4):e01545-e1622. 35856669 10.1128/spectrum.01545-22 PMC9430409

[155] Ali Z, Aman R, Mahas A, Rao GS, Tehseen M, Marsic T, et al. iSCAN: An RT-LAMP-coupled CRISPR-Cas12 module for rapid, sensitive detection of SARS-CoV-2. Virus Res. 2020;288:198129. 32822689 10.1016/j.virusres.2020.198129 PMC7434412

[156] Mollasalehi H, Vahedipour N, Taghvamanesh A, Minai-Tehrani D. Development of one-pot single specific primer-LAMP (SSP-LAMP) for identification of Shigella genus using 16S rDNA. Anal Biochem. 2022;636:114452. 34762873 10.1016/j.ab.2021.114452

[157] Chen S, Wu C, Qian C, Pang Y, Guo K, Wang T, et al. Ultraspecific one-pot CRISPR-based “Green-Yellow-Red” multiplex detection strategy integrated with portable cartridge for point-of-care diagnosis. Anal Chem. 2024;96(7):3145–52. 10.1021/acs.analchem.3c05493 38324761

[158] Li Q-N, Wang D-X, Chen D-Y, Lyu J-A, Wang Y-X, Wu S-L, et al. Photoactivatable CRISPR/Cas12a sensors for biomarkers imaging and point-of-care diagnostics. Anal Chem. 2024;96(6):2692–701. 38305871 10.1021/acs.analchem.3c05497

[159] Hu M, Qiu Z, Bi Z, Tian T, Jiang Y, Zhou X. Photocontrolled crRNA activation enables robust CRISPR-Cas12a diagnostics. Proc Natl Acad Sci. 2022;119(26):e2202034119. 35727982 10.1073/pnas.2202034119 PMC9245704

[160] Suea-Ngam A, Howes PD, deMello AJ. An amplification-free ultra-sensitive electrochemical CRISPR/Cas biosensor for drug-resistant bacteria detection. Chem Sci. 2021;12(38):12733–43. 34703560 10.1039/d1sc02197d PMC8494034

[161] Shi K, Xie S, Tian R, Wang S, Lu Q, Gao D, et al. A CRISPR-Cas autocatalysis-driven feedback amplification network for supersensitive DNA diagnostics. Sci Adv. 2021;7(5):eabc7802. 33571114 10.1126/sciadv.abc7802 PMC7840123

[162] Wei C, Lei X, Yu S. Multiplexed detection strategies for biosensors based on the CRISPR-Cas system. ACS Synth Biol. 2024;13(6):1633–46. 38860462 10.1021/acssynbio.4c00161

[163] Patchsung M, Homchan A, Aphicho K, Suraritdechachai S, Wanitchanon T, Pattama A, et al. A multiplexed Cas13-based assay with point-of-care attributes for simultaneous COVID-19 diagnosis and variant surveillance. Crispr J. 2023;6(2):99–115. 36367987 10.1089/crispr.2022.0048 PMC7614457

[164] Ackerman CM, Myhrvold C, Thakku SG, Freije CA, Metsky HC, Yang DK, et al. Massively multiplexed nucleic acid detection with Cas13. Nature. 2020;582(7811):277–82. 32349121 10.1038/s41586-020-2279-8 PMC7332423

[165] Welch NL, Zhu M, Hua C, Weller J, Mirhashemi ME, Nguyen TG, et al. Multiplexed CRISPR-based microfluidic platform for clinical testing of respiratory viruses and identification of SARS-CoV-2 variants. Nat Med. 2022;28(5):1083–94. 35130561 10.1038/s41591-022-01734-1 PMC9117129

[166] Fozouni P, Son S, Díaz de León Derby M, Knott GJ, Gray CN, D’Ambrosio MV, et al. Amplification-free detection of SARS-CoV-2 with CRISPR-Cas13a and mobile phone microscopy. Cell. 2021;184(2):323–3319. 33306959 10.1016/j.cell.2020.12.001 PMC7834310

[167] Silva FSR, Erdogmus E, Shokr A, Kandula H, Thirumalaraju P, Kanakasabapathy MK, et al. SARS-CoV-2 RNA detection by a cellphone-based amplification-free system with CRISPR/CAS-dependent enzymatic (CASCADE) assay. Adv Mater Technol. 2021;6(12):2100602. 34514084 10.1002/admt.202100602 PMC8420437

[168] Samacoits A, Nimsamer P, Mayuramart O, Chantaravisoot N, Sitthi-amorn P, Nakhakes C, et al. Machine learning-driven and smartphone-based fluorescence detection for CRISPR diagnostic of SARS-CoV-2. ACS Omega. 2021;6(4):2727–33. 33553890 10.1021/acsomega.0c04929 PMC7839157

[169] Huang D, Shi Z, Qian J, Bi K, Fang M, Xu Z. A CRISPR-Cas12a-derived biosensor enabling portable personal glucose meter readout for quantitative detection of SARS-CoV-2. Biotechnol Bioeng. 2021;118(4):1587–96. 33410130 10.1002/bit.27673

[170] Zhou C, Huang D, Wang Z, Shen P, Wang P, Xu Z. CRISPR Cas12a-based “sweet” biosensor coupled with personal glucose meter readout for the point-of-care testing of Salmonella. J Food Sci. 2022;87(9):4137–47. 35986652 10.1111/1750-3841.16287

